# Factors predicting prolonged empirical antifungal treatment in critically ill patients

**DOI:** 10.1186/1476-0711-13-11

**Published:** 2014-03-11

**Authors:** Mohamed Zein, Erika Parmentier-Decrucq, Amer Kalaoun, Olivier Bouton, Frédéric Wallyn, Anne Baranzelli, Dia Elmanser, Boualem Sendid, Saad Nseir

**Affiliations:** 1 Pôle de Réanimation Médicale, CHRU de Lille, Hôpital Salengro, Lille, France; 2 Laboratoire de Mycologie et de Parasitologie, CHRU de Lille, Lille, France; 3 Université Lille Nord de France, Lille, France

**Keywords:** Antifungal treatment, Empirical treatment, Fungal infection, Invasive fungal disease, De-escalation

## Abstract

**Objective:**

To determine the incidence, risk factors, and impact on outcome of prolonged empirical antifungal treatment in ICU patients.

**Methods:**

Retrospective observational study performed during a one-year period. Patients who stayed in the ICU >48 h, and received empirical antifungal treatment were included. Patients with confirmed invasive fungal disease were excluded. Prolonged antifungal treatment was defined as percentage of days in the ICU with antifungals > median percentage in the whole cohort of patients.

**Results:**

Among the 560 patients hospitalized for >48 h, 153 (27%) patients received empirical antifungal treatment and were included in this study. Fluconazole was the most frequently used antifungal (46% of study patients). Median length of ICU stay was 19 days (IQR 8, 34), median duration of antifungal treatment was 8 days (IQR 3, 16), and median percentage of days in the ICU with antifungals was 48% (IQR 25, 80). Seventy-seven patients (50%) received prolonged empirical antifungal treatment. Chemotherapy (OR [95% CI] 2.6 [1.07-6.69], p = 0.034), and suspected infection at ICU admission (3.1 [1.05-9.48], p = 0.041) were independently associated with prolonged empirical antifungal treatment.

Duration of mechanical ventilation and ICU stay were significantly shorter in patients with prolonged empirical antifungal treatment compared with those with no prolonged empirical antifungal treatment. However, ICU mortality was similar in the two groups (46 versus 52%, p = 0.62).

**Conclusion:**

Empirical antifungal treatment was prescribed in a large proportion of study patients. Chemotherapy, and suspicion of infection at ICU admission are independently associated with prolonged empirical antifungal treatment.

## Introduction

Invasive fungal disease is common in critically ill patients. Based on the results of the large international EPIC II study [1], fungi are responsible for 19.4% of all documented infections in the intensive care unit (ICU). These patients have several risk factors for fungal infection, including invasive procedures, prolonged antimicrobial treatment, fungal colonization, abdominal surgery, parental nutrition, and immunosuppression [2,3]. Substantial morbidity and mortality were reported in ICU patients with confirmed invasive fungal disease. Prompt appropriate antifungal treatment is a key factor in the prognosis of ICU patients suffering from invasive fungal disease [4]. Therefore, empirical antifungal treatment is frequently prescribed to these patients when fungal infection is suspected. However, the diagnosis of invasive fungal disease is still extremely difficult in this population, because of the absence of an accurate non invasive diagnostic method [5]. In addition, no clear evidence-based recommendations are available on how and when to de-escalate antifungal treatment. Therefore, empirical antifungal treatment is frequently used for a long period of time in the ICU. Prolonged antifungal treatment is responsible for higher hospital cost, and might promote antifungal resistance.

To our knowledge, few data are available on prolonged empirical antifungal treatment. A recent study was conducted to determine the incidence of systemic antifungal use in critically ill patients without invasive fungal disease [6]. However, no information was provided on prolonged empirical antifungal treatment incidence. In addition, risk factors for prolonged duration of antifungal treatment and its impact on outcome are unknown. Further, prolonged antifungal use is a well-known risk factor for emergence of antifungal resistance [7]. Identifying the incidence, risk factors and impact on outcome of prolonged empirical antifungal treatment might be helpful for future studies aiming at reducing the duration of this treatment. Therefore, we conducted this retrospective observational study to determine, incidence, risk factors, and impact on outcome of prolonged antifungal treatment.

## Patients and methods

This retrospective study was conducted in a 30-bed medical and surgical university ICU during a one-year period (from January 2011 to January 2012). Informed consent was not required by the local IRB because of the retrospective non interventional design of the study.

All adult patients hospitalized in the ICU for more than 48 hours who received empirical antifungal treatment were eligible for this study. Patients with confirmed invasive fungal disease were excluded.

### Study patients

In patients with suspected invasive fungal infection, blood cultures were systematically performed before empirical antifungal treatment [8]. In addition, other microbiological specimens were performed based on patient’s clinical status. Candida colonization index was not routinely performed. Antifungal treatment was based on written local guidelines. Briefly, fluconazole, or caspofungin were recommended as initial therapy for suspected invasive candidiasis in non neutropenic patients. Lipid formulation of amphotericin B was recommended as alternative if there is intolerance to other antifungals. In neutropenic patients, lipid formulation of amphotericin B, caspofungin, or voriconazole were recommended for empirical treatment of suspected candidiasis. No prophylactic antifungal treatment was used during the study period.

### Definitions

Prolonged antifungal treatment was defined as percentage of days in the ICU with antifungals > median percentage in the whole cohort of patients. Recent hospitalization was defined as any hospitalization for >48 h during the 3 months preceding ICU admission. Immunosuppression was defined as neutropenia (<500/μL), chemotherapy during the last three months, or long-term corticosteroids use. Prior antimicrobial, and antifungal treatment, was defined as any treatment by these agents during the 3 months preceding ICU admission.

### Data collection

The following data were collected at ICU admission: age, gender, simplified acute physiology score II, logistic organ dysfunction score, location before ICU admission (other wards, other ICUs, home), recent hospitalization, category of admission (medical, surgical, trauma), comorbidities (diabetes mellitus, chronic heart failure, chronic obstructive pulmonary disease, cirrhosis, chronic dialysis, immunosuppression), McCabe score, causes for ICU admission, suspected infection at ICU admission, prior antibiotic treatment, and prior antifungal treatment. In addition, the following data were collected during ICU stay: number of sites colonized with fungi, antibiotic treatment, mechanical ventilation, central venous catheter, arterial catheter, dialysis, duration of ICU stay, duration of antifungal treatment, parental nutrition, and ICU mortality.

### Statistical analysis

The percentage of days in the ICU with antifungal treatment was calculated for each patient, as well as median (25^th^, 75^th^ IQR) percentage in the study cohort. Patients were classified as having a prolonged duration of antifungal treatment if they had a percentage of days in the ICU with antifungal treatment > median percentage of days in the ICU with antifungals, or as not having a prolonged duration of antifungal treatment if they had a percentage of days in the ICU with antifungal treatment ≤ median percentage of days in the ICU with antifungals.

Statistical analysis was performed using SPSS software (version 15.0, SPSS, Chicago, IL). Results of qualitative variables are presented as numbers (percentage). Distribution of quantitative variables was tested. Normally and non normally distributed variables are expressed as mean ± SD and median (25^th^, 75^th^ IQR); respectively.

To determine risk factors for prolonged empirical antifungal treatment, patients with prolonged empirical antifungal treatment were compared with patients with no prolonged empirical antifungal treatment by univariate analysis. Qualitative variables were compared using chi2 or Fischer’s exact test, as appropriate. Quantitative variables were compared using Student’s t test or Mann Whitney U test, as appropriate. Multivariate analysis was used to determine risk factors independently associated with prolonged empirical antifungal treatment. All data from univariate analysis with *p* values <0.1 were incorporated in the multivariate conditional logistic regression analysis. Backward stepwise was used in the logistic regression model. The Hosmer-Lemeshow test was used to determine the model goodness-of-fit.

## Results

**Figure 1:**
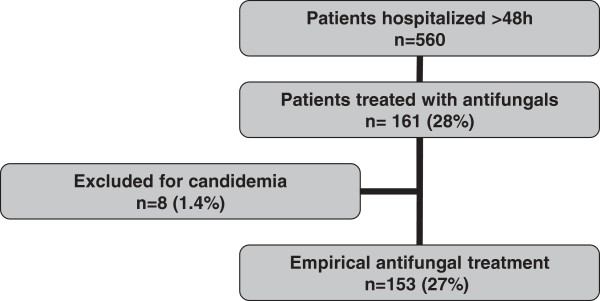
Study flowchart.

Among the 560 patients hospitalized >48 h during the study period, 161 (28%) patients were eligible for this study, and 153 (27%) patients were included. Eight (1.4%) patients were excluded for confirmed invasive fungal disease (8 candidemia) (Figure 1). Fluconazole was the most frequently used antifungal (46% of study patients), followed by caspofungin (39%), voriconazole (12%), and liposomal amphotericin B (3%). Four patients (2%) received more than one antifungal during their ICU stay.

In study patients, median length of ICU stay was 19 days (IQR 8, 34), median duration of antifungal treatment was 8 days (IQR 3, 16), and median percentage of days in the ICU with antifungals was 48% (IQR 25, 80). Seventy-seven patients (50%) received prolonged empirical antifungal treatment. Patient characteristics are presented in Tables 1, and 2. 35 patients (22% of study patients, and 6% of the whole cohort) received empirical antifungal treatment before ICU admission.

**Table 1:** Risk factors for prolonged empirical antifungal treatment at ICU admission

**Patient characteristics**	**Prolonged empirical antifungal treatment**		**p value**
	**Yes n = 77**	**No n = 76**	
Age, years, mean ± SD	55 ± 14	60 ± 15	0.429
Male	42 (54)	49 (64)	0.277
SAPS II	54 (35, 71)	52 (37, 73)	0.518
LOD score	7 (4,10)	7.5 (4,11)	0.423
Location before ICU admission			0.963
Other wards	60 (78)	58 (76)	
Other ICUs	7 (9)	7 (9)	
Home	10 (13)	11 (14)	
Recent hospitalization	34 (44)	22 (28)	0.058
Category of admission			0.500
Medical	58 (75)	51 (67)	
Surgical	18 (23)	23 (30)	
Trauma	1 (1)	2 (2)	
Comorbidities			
Diabetes	14 (18)	19 (25)	0.305
Chronic heart failure	15 (19)	9 (12)	0.192
COPD	15 (19)	16 (21)	0.809
Cirrhosis	4 (5)	4 (5)	>0.999
Chronic dialysis	6 (7)	5 (6)	0.771
Immunosuppression			
Neutropenia	14 (18)	7 (9)	0.111
Chemotherapy	22 (28)	8 (10)	0.005*
Corticosteroids	21 (27.3)	9 (12)	0.017*
McCabe score			0.137
Non fatal disease	24 (31)	34 (44)	
Ultimately fatal disease	23 (29)	14 (18)	
Rapidly fatal disease	30 (38)	28 (36)	
Causes for ICU admission**			
Septic shock	42 (54)	36 (47)	0.374
Acute exacerbation of COPD	21 (27)	14 (18)	0.192
ARDS	13 (16)	20 (26)	0.156
CAP	11 (14)	14 (18)	0.489
HAP	24 (31)	12 (15)	0.024*
Congestive heart failure	5 (6)	2 (2)	0.442
Poisoning	2 (2)	8 (10)	0.056
Neurologic failure	2 (2)	6 (7)	0.167
Infection on admission	72 (93)	58 (76)	0.002*
Prior antibiotic treatment	45 (58)	33 (43)	0.063
Prior antifungal treatment	25 (32.5)	10 (13)	0.004*

*OR (95% CI) 3.3 (1.3-8.1), 2.7 (1.1-6.4), 2.4 (1.1-5.2), 4.4 (1.5-12.7), 3.1 (1.4-7.1); respectively.

**Several patients had more than one cause for ICU admission.

Data are n (%), or median (25^th^, 75^th^ IQR), unless otherwise specified.

SAPS, simplified acute physiology score; LOD, logistic organ dysfunction; ICU, intensive care unit; COPD, chronic obstructive pulmonary disease; ARDS, acute respiratory distress syndrome; CAP, community acquired pneumonia; HAP, hospital acquired pneumonia.

**Table 2:** Patient characteristics during ICU stay

**Patient characteristics**	**Prolonged empirical antifungal treatment**		**p value**
	**Yes n = 77**	**No n = 76**	
Multifocal fungal colonization	24 (31)	35 (46)	0.076
Antimicrobial treatment	73 (94)	70 (93)	0.744
Mechanical ventilation	59 (76)	67 (88)	0.059
Duration of mechanical ventilation, d	12.5 (4, 25)	20 (0, 36)	0.007
Central venous catheter	69 (89)	71 (93)	0.396
Duration of central venous catheter use, d	13 (4, 20)	18 (10, 25)	0.015
Arterial catheter	62 (81)	71 (93)	0.025*
Duration of arterial catheter use, d	8 (3, 15)	13 (10, 20)	0.002
Dialysis	30 (39)	28 (37)	0.97
Parenteral nutrition	26 (33)	29 (38)	0.571
Duration of antifungal treatment, d	9 (4, 19)	6 (2, 11)	0.003
Percentage of days in the ICU with antifungals	80 (65, 100)	25 (13, 35)	<0.001
Length of ICU stay, d	14 (5, 27)	22 (14, 39)	0.001
ICU mortality	36 (46)	39 (52)	0.518

*OR (95% CI) 0.3 (0.1-0.9).

Data are n (%), or median (25^th^, 75^th^ IQR).

ICU, intensive care unit.

### Factors associated with prolonged empirical antifungal treatment by univariate analysis

At ICU admission, chemotherapy, corticosteroid use, hospital-acquired pneumonia, suspected infection, and prior antifungal treatment were significantly more frequent in patients with prolonged antifungal treatment compared with those with no prolonged antifungal treatment.

During ICU stay, percentage of patients with arterial catheter, duration of central venous catheter use, and arterial catheter use were significantly lower in patients with prolonged antifungal treatment compared with those with no prolonged antifungal treatment.

### Factors associated with prolonged empirical antifungal treatment by multivariate analysis

Chemotherapy (OR [95% CI] 2.6 [1.07-6.69], p = 0.034), and infection at ICU admission (3.1 [1.05-9.48], p = 0.041) were independently associated with prolonged empirical antifungal treatment. Hosmer-Lemeshow test was not significant (p 0.579), confirming the model goodness-of-fit.

### Impact of prolonged empirical antifungal treatment on outcome

Duration of mechanical ventilation (12.5 [4,9] versus 20 d (0, 36), p = 0,007) and ICU stay (18 [5-28] versus 24 d [15–38], p = 0.001) were significantly shorter in patients with prolonged empirical antifungal treatment compared with those with non prolonged empirical antifungal treatment. However, ICU mortality was similar in the two groups (46 versus 52%, p = 0.62).

## Discussion

Our results suggest that empirical antifungal treatment is used in about one third of ICU patients hospitalized for more than 48 h, during 48% of their ICU stay. Chemotherapy, and suspicion of infection at ICU admission were independently associated with prolonged empirical antifungal treatment. Although duration of mechanical ventilation and ICU stay were significantly shorter in patients with prolonged empirical antifungal treatment compared with those with no prolonged empirical antifungal treatment, no significant impact of prolonged empirical antifungal treatment was found on ICU mortality.

To the best of our knowledge, our study is the first to report on the incidence of prolonged use of empirical antifungal treatment. Empirical antifungal treatment was used in a large percentage (27%) of ICU patients, during a large percentage (48%) of their ICU stay. A recent Spanish multicenter study [9] analyzed 8240 antifungal prescriptions during a 5 year-period. An increase in antifungal use was reported to the year 2008, with a subsequent stabilization. The median duration of antifungal treatment was 8 days, which is in line with our results. However, no information was provided on empirical antifungal use, or on duration of ICU stay. Another recent one-day cross sectional multicenter study was performed to determine the incidence of ICU patients without documented invasive fungal disease who receive systematic antifungal treatment [6]. Among the 2047 included patients, 5.2% received antifungal treatment without confirmed invasive fungal disease. At least two reasons could explain the lower rate of empirical antifungal use found in this study compared with ours. First, this study was a one day study. Therefore, antifungals stopped before, or started after the study’s day were not taken into account. Second, patients with short duration (<48H) of ICU stay were not excluded from that study. These patients rarely receive antifungal treatment.

Chemotherapy, and suspicion of infection at ICU admission were identified as independent risk factors for prolonged empirical antifungal use. These factors might reflect patients with increased risk for invasive fungal disease. Previous studies reported that immunosuppressed patients, and those with other infections and prior antibiotic treatment are at higher risk for invasive fungal disease [10-13]. However, none of these factors is modifiable.

There is no consensual definition for prolonged antifungal treatment, probably because this duration could be very different according to ICU population and local protocols. In order to adjust for duration of ICU stay, and to take into account the number of days in the ICU without antifungals, we used the median of percentage of days in the ICU with antifungals to define prolonged antifungal treatment. This definition is probably more accurate than total duration of antifungal treatment, or days in the ICU without antifungals. For example, two patients who receive the same duration of antifungal treatment (7 days) in the ICU could have a percentage of days in the ICU with antifungals of 100% and 50%, if they are discharged at day 7, and day 14; respectively. This adjustment was already used for antimicrobials in previous studies [14,15].

Duration of mechanical ventilation and ICU stay were significantly shorter in patients with prolonged empirical antifungal treatment compared with those with no prolonged empirical antifungal treatment. This result could be explained by the definition we used for prolonged antifungal treatment. Based on this definition, the shorter duration of ICU stay, the higher percentage of days with empirical antifungal treatment. However, no significant impact of prolonged empirical antifungal treatment was found on ICU mortality, suggesting that prolonged empirical antifungal treatment is probably not justified in this population. Further, percentage of patients with arterial catheter, and those under mechanical ventilation are higher in patients with no prolonged empirical antifungal treatment compared with those with prolonged antifungal treatment. The explanation for these findings is probably the longer duration of ICU stay in patients with no prolonged duration of antifungal treatment. Previous studies clearly showed that patients with longer duration of ICU stay receive more invasive procedures, for longer period of time [16,17].

Potential explanations for the high percentage of patients with prolonged antifungal treatment in our ICU include the difficult diagnosis of invasive fungal disease in ICU patients [18], and the absence of recommendations on optimal duration of treatment and de-escalation in this population [5]. Prolonged use of antifungals is associated with high hospital cost [19], and increased risk for fungal resistance [7]. Further studies should determine the impact of clinical, and biological markers use on duration of empirical antifungal treatment, and patient outcomes. For example, the use of PCR [20,21], galactomannan [22], and biomarkers for invasive fungal disease, such as mannan, anti-mannan [9,23,24], and β-D-glucan [25-29], could be helpful for reducing duration of empirical antifungal treatment. In our ICU, galactomannan, and biomarkers for invasive fungal disease are available, and used by physicians. However, no clear recommendation is available on how to use these markers to tailor antifungal treatment initiation or duration in critically ill patients.

Our study has some limitations, including the retrospective observational design, and the single center design. Therefore, other large prospective multicenter studies are needed to confirm our results. Further, no information was collected on corticosteroid treatment during ICU stay. In fact, corticosteroids treatment in the ICU might reflect refractory septic shock. No significant difference was found in percentage of patients with septic shock at ICU admission between patients with prolonged empirical antifungal use and those with no prolonged empirical treatment. However, no information was collected on septic shock occurring during ICU stay. In addition, as no information was available on appropriateness of antibiotic treatment in patients with infection, we could not adjust for for this factor in mortality analysis. Finally, as explained above, the prolonged duration of mechanical ventilation and ICU stay in patients who had no prolonged duration of antifungal treatment is probably due to the definition we used for this condition. However, the possibility of beneficial effects of antifungal treatment on duration of mechanical ventilation and ICU stay could not be ruled out. Further randomized controlled studies are needed to determine the impact of duration of empirical antifungal treatment on outcome of critically ill patients.

## Conclusion

Empirical antifungal treatment was used in a large proportion of study patients. Chemotherapy, and suspicion of infection at ICU admission were independently associated with prolonged empirical antifungal treatment. No significant impact of prolonged empirical antifungal treatment was found on ICU mortality.

## Competing interests

The authors declare that they have no competing interests.

## Authors’ contributions

EP, BS, and SN designed the study. MZ, AK, OB, FW, AB, FW, and DE collected the data. MZ, EP, and SN drafted the manuscript. All authors read and approved the final manuscript.

## References

[B1] Vincent J-L Rello J Marshall J Silva E Anzueto A Martin CD Moreno R Lipman J Gomersall C Sakr Y Reinhart K International study of the prevalence and outcomes of infection in intensive care units JAMA 2009 13 2323 9 10.1001/jama.2009.1754 19952319

[B2] Ostrosky-Zeichner L Invasive mycoses: diagnostic challenges Am J Med 2012 13 S14 24 22196205 10.1016/j.amjmed.2011.10.008

[B3] Eggimann P Bille J Marchetti O Diagnosis of invasive candidiasis in the ICU Ann Intensive Care 2011 13 37 10.1186/2110-5820-1-37 21906271 PMC3224461

[B4] Cornely OA Bassetti M Calandra T Garbino J Kullberg BJ Lortholary O Meersseman W Akova M Arendrup MC Arikan-Akdagli S Bille J Castagnola E Cuenca-Estrella M Donnelly JP Groll AH Herbrecht R Hope WW Jensen HE Lass-Flörl C Petrikkos G Richardson MD Roilides E Verweij PE Viscoli C Ullmann AJ ESCMID* guideline for the diagnosis and management of Candida diseases 2012: non-neutropenic adult patients Clin Microbiol Infect 2012 13 Suppl 7 19 37 23137135 10.1111/1469-0691.12039

[B5] Ostrosky-Zeichner L Kullberg BJ Bow EJ Hadley S León C Nucci M Patterson TF Perfect JR Early treatment of candidemia in adults: a review Med Mycol 2011 13 113 20 10.3109/13693786.2010.512300 20818922

[B6] Azoulay E Dupont H Tabah A Lortholary O Stahl J-P Francais A Martin C Guidet B Timsit J-F Systemic antifungal therapy in critically ill patients without invasive fungal infection Crit Care Med 2012 13 813 22 10.1097/CCM.0b013e318236f297 22297630

[B7] Fournier P Schwebel C Maubon D Vesin A Lebeau B Foroni L Hamidfar-Roy R Cornet M Timsit J-F Pelloux, H Antifungal use influences Candida species distribution and susceptibility in the intensive care unit J Antimicrob Chemoth 2011 13 2880 6 10.1093/jac/dkr394 21980066

[B8] Schuster MG Edwards JE Sobel JD Darouiche RO Karchmer AW Hadley S Slotman G Panzer H Biswas P Rex JH Empirical fluconazole versus placebo for intensive care unit patients: a randomized trial Ann Intern Med 2008 13 83 90 10.7326/0003-4819-149-2-200807150-00004 18626047

[B9] Olaechea-Astigarraga PM Alvarez-Lerma F Palomar-Martínez M Insausti-Ordeñana J López-Pueyo MJ Seijas-Betolaza I Otal-Entraigas JJ Gimeno-Costa R Gracia-Arnillas MP Trends in systemic antifungal use in critically ill patients. Multicenter observational study, 2006–2010 Enfermedades infecciosas Microbiología Clínica 2012 13 435 40 10.1016/j.eimc.2012.02.006 22463989

[B10] Yang S-P Chen Y-Y Hsu H-S Wang F-D Chen L Fung C-P A risk factor analysis of healthcare-associated fungal infections in an intensive care unit: a retrospective cohort study BMC Infect Dis 2013 13 10 10.1186/1471-2334-13-10 23298156 PMC3548709

[B11] Pratikaki M Platsouka E Sotiropoulou C Douka E Paramythiotou E Kaltsas P Kotanidou A Paniara O Roussos C Routsi C Epidemiology, risk factors for and outcome of candidaemia among non-neutropenic patients in a Greek intensive care unit Mycoses 2011 13 154 61 10.1111/j.1439-0507.2009.01787.x 19793354

[B12] Vardakas KZ Michalopoulos A Kiriakidou KG Siampli EP Samonis G Falagas ME Candidaemia: incidence, risk factors, characteristics and outcomes in immunocompetent critically ill patients Clin Microbiol Infect 2009 13 289 92 10.1111/j.1469-0691.2008.02653.x 19154488

[B13] León C Alvarez-Lerma F Ruiz-Santana S León MA Nolla J Jordá R Saavedra P Palomar M Fungal colonization and/or infection in non-neutropenic critically ill patients: results of the EPCAN observational study Eur J Clin Microbiol Infect Dis 2009 13 233 42 10.1007/s10096-008-0618-z 18758831

[B14] Drees M Snydman DR Schmid CH Barefoot L Hansjosten K Vue PM Cronin M Nasraway SA Golan Y Golan, Y Prior environmental contamination increases the risk of acquisition of vancomycin-resistant enterococci Clin Infect Dis 2008 13 678 85 10.1086/527394 18230044

[B15] Nseir S Zerimech F Fournier C Lubret R Ramon P Durocher A Balduyck M Continuous control of tracheal cuff pressure and microaspiration of gastric contents in critically ill patients Am J Respir Crit Care Med 2011 13 1041 7 10.1164/rccm.201104-0630OC 21836137

[B16] Van der Kooi TII de Boer AS Manniën J Wille JC Beaumont MT Mooi BW van den Hof S Incidence and risk factors of device-associated infections and associated mortality at the intensive care in the Dutch surveillance system Intensive Care Med 2007 13 271 8 10.1007/s00134-006-0464-3 17146632

[B17] Nseir S Hoel J Grailles G Soury-Lavergne A Di Pompeo C Mathieu D Durocher A Remifentanil discontinuation and subsequent intensive care unit-acquired infection: a cohort study Crit Care 2009 13 R60 10.1186/cc7788 19383164 PMC2689508

[B18] Monneret G Venet F Kullberg B-J Netea MG ICU-acquired immunosuppression and the risk for secondary fungal infections Med Mycol 2011 13 Suppl 1 S17 23 20718607 10.3109/13693786.2010.509744

[B19] Dodds Ashley E Drew R Johnson M Danna R Dabrowski D Walker V Prasad M Alexander B Papadopoulos G Perfect J Cost of invasive fungal infections in the era of new diagnostics and expanded treatment options Pharmacotherer 2012 13 890 901 10.1002/j.1875-9114.2012.01124 23033228

[B20] Lass-Flörl C Mutschlechner W Aigner M Grif K Marth C Girschikofsky M Grander W Greil R Russ G Cerkl P Eller M Kropshofer G Eschertzhuber S Kathrein H Schmid S Beer R Lorenz I Theurl I Nachbaur D Utility of PCR in diagnosis of invasive fungal infections: real-life data from a multicenter study J Clin Microbiol 2013 13 863 8 10.1128/JCM.02965-12 23269732 PMC3592065

[B21] Nguyen MH Wissel MC Shields RK Salomoni MA Hao B Press EG Shields RM Cheng S Mitsani D Vadnerkar A Silveira FP Kleiboeker SB Clancy CJ Performance of Candida real-time polymerase chain reaction, β-D-glucan assay, and blood cultures in the diagnosis of invasive candidiasis Clin Infect Dis 2012 13 1240 8 10.1093/cid/cis200 22431804

[B22] Morrissey CO Chen SC-A Sorrell TC Milliken S Bardy PG Bradstock KF Szer J Halliday CL Gilroy NM Moore J Schwarer AP Guy S Bajel A Tramontana AR Spelman T Slavin MA Galactomannan and PCR versus culture and histology for directing use of antifungal treatment for invasive aspergillosis in high-risk haematology patients: a randomised controlled trial Lancet Infect Dis 2013 13 519 28 10.1016/S1473-3099(13)70076-8 23639612

[B23] Mikulska M Calandra T Sanguinetti M Poulain D Viscoli C The use of mannan antigen and anti-mannan antibodies in the diagnosis of invasive candidiasis: recommendations from the Third European Conference on Infections in Leukemia Crit Care 2010 13 R222 10.1186/cc9365 21143834 PMC3219989

[B24] Sendid B Dotan N Nseir S Savaux C Vandewalle P Standaert A Zerimech F Guery BP Dukler A Colombel JF Poulain D Antibodies against glucan, chitin, and Saccharomyces cerevisiae mannan as new biomarkers of Candida albicans infection that complement tests based on C. albicans mannan Clin Vaccine Immunol 2008 13 1868 77 10.1128/CVI.00200-08 18971303 PMC2593178

[B25] León C Ruiz-Santana S Saavedra P Castro C Ubeda A Loza A Martín-Mazuelos E Blanco A Jerez V Ballús J Alvarez-Rocha L Utande-Vázquez A Fariñas O Value of β-D-glucan and Candida albicans germ tube antibody for discriminating between Candida colonization and invasive candidiasis in patients with severe abdominal conditions Intensive Care Med 2012 13 1315 25 10.1007/s00134-012-2616-y 22752333

[B26] Fontana C Gaziano R Favaro M Casalinuovo I Pistoia E Di Francesco P (1–3)-β-D-Glucan vs Galactomannan Antigen in Diagnosing Invasive Fungal Infections (IFIs) Open Microbiol J 2012 13 70 3 10.2174/1874285801206010070 22942923 PMC3431562

[B27] Hanson KE Pfeiffer CD Lease ED Balch AH Zaas AK Perfect JR Alexander BD β-D-glucan surveillance with preemptive anidulafungin for invasive candidiasis in intensive care unit patients: a randomized pilot study PloS One 2012 13 e42282 10.1371/journal.pone.0042282 22879929 PMC3412848

[B28] Tissot F Lamoth F Hauser PM Orasch C Flückiger U Siegemund M Zimmerli S Calandra T Bille J Eggimann P Marchetti O Beta-Glucan Antigenemia Anticipates Diagnosis of Blood Culture-Negative Intra-Abdominal Candidiasis Am J Respir Crit Care Med 2013 doi:10.1164/rccm.201211-2069OC 10.1164/rccm.201211-2069OC 23782027

[B29] Posteraro B De Pascale G Tumbarello M Torelli R Pennisi MA Bello G Maviglia R Fadda G Sanguinetti M Antonelli M Early diagnosis of candidemia in intensive care unit patients with sepsis: a prospective comparison of (1 → 3)-β-D-glucan assay, Candida score, and colonization index Crit Care 2011 13 R249 10.1186/cc10507 22018278 PMC3334800

